# The Influence of Multi-morbidity on the Work Ability of Ageing Employees and the Role of Coping Style

**DOI:** 10.1007/s10926-018-9811-9

**Published:** 2018-09-03

**Authors:** Emelien A. Kadijk, Swenneke van den Heuvel, Jan Fekke Ybema, Fenna R. M. Leijten

**Affiliations:** 1 Erasmus School of Health Policy & Management, Erasmus University Rotterdam, P.O.Box 1738, 3000 DR Rotterdam, The Netherlands; 2 Netherlands Organisation for Applied Scientific Research TNO, Schipholweg 77, 2316 ZL Leiden, The Netherlands; 3 Faculty of Social and Behavioural Sciences, Utrecht University, Heidelberglaan 1, 3581 CS Utrecht, The Netherlands

## Abstract

*Purpose* With an ageing workforce, employees are increasingly confronted with multi-morbidity. Especially physical and mental health problems often occur together. This study aims to (i) explore the effect of multi-morbidity on work ability of ageing employees, more specifically the effects of the number of health problems and the combination of physical and mental health problems, and to (ii) explore to what extent the effects of physical and mental health problems on work ability are explained by applying differing coping styles. *Methods* A 1 year follow up study (2012–2013) was conducted among 7175 employees aged 45–64 years. Linear regression analyses were conducted to examine longitudinal relationships between multi-morbidity, coping styles and work ability. To determine whether coping styles mediate the effects of multi-morbidity on work ability, Sobel tests were conducted. *Results* A higher number of health problems was related to poorer work ability, but this negative effect stabilized from three health problems onwards. The combination of physical and mental health problem(s) was more strongly related to poorer work ability than only physical health problems. The negative relation between physical health problems and work ability was partly suppressed by active coping, while the negative relation between the combination of physical and mental health problem(s) on work ability was partly explained by avoidant coping. *Conclusions* Ageing employees with multi-morbidity have a reduced work ability, especially when mental health problems are present. The greater negative effects of the combination of physical and mental health problems on work ability are partially due to unfavorable coping styles.

## Background

In order to be able to fund ageing societies, it is inevitable that employees must work longer. However, older employees often suffer from chronic health problems. In the period from 2007 to 2011, approximately 37% of the Dutch working population annually reported that they suffer from one or more chronic health problem [1]. Furthermore, 13% of the population and 37% of persons older than 55 suffer from multi-morbidity, defined as the co-existence of more than one chronic health problem [2]. The prevalence of multi-morbidity is expected to continue to grow, mainly caused by the increasing life expectancy, unhealthy lifestyle and improved treatment, which leads to increasing survival rates of individuals with chronic health problems [3].

Especially physical and mental health problems occur together more often than can be expected based on coincidence [4–6]. This may occur for several reasons. First, physical health problems carry the risk of pain and disability that can lead to mental health problems [7]. Second, mental health problems are associated with a poor lifestyle [8] and with abusing alcohol and drugs [9] which may induce physical health consequences. Third, research shows that shared vulnerabilities may be partly responsible for the occurrence of mental and physical health problems. It appears that high anxiety sensitivity is associated with both mental health problems and physical problems [10]. Further, avoidance behaviors and avoidant coping styles are associated with more mental and physical health problems [11–15]. Fourth, fragmented health care might contribute to a belated detection of health problems. Especially mental health care has traditionally been segregated from other medical disciplines, expressed for example by the geographic separation of mental health institutions from other care providers [3]. Persons with multi-morbidities that require care across these different sectors may thus not receive optimal care, which could in turn worsen their health situation.

Older persons with an increased likelihood of having health problems are in turn likely to have a reduced work ability [16–18]. Work ability is defined as the degree to which an employee is both physically as well as mentally able to work, given his or her resources (e.g., health, functional abilities, competencies) and work demands (e.g., work environment, contents, demands) [19, 20]. Low work ability is associated with early retirement [21, 22] and incapacity for work [23]. Considering the high prevalence of multi-morbidity, more insight is needed into the effects thereof on work ability amongst older workers. Therefore, the first aim in this study is to investigate the relationship between multi-morbidity and work ability. We use four definitions of multi-morbidity in this study, all of which are based on literature. In most studies researching the effects of multi-morbidity on employment outcomes, the number of health problems is grouped (i.e., single vs. two or more health problems). This makes determining the impact of the specific number of health problems unclear. For this reason, in the current study, we examine whether an increase of the number of health problems leads to a decrease in work ability and at what point an additional health problem no longer negatively impacts work ability (*definition i*).

In addition, research shows that mental health problems often have more negative effects on health-related outcomes than physical health problems [24]. This study examines whether this is also the case if work ability is the outcome of interest (*definition ii*).

Further, multi-morbidity is defined in literature as having two or more chronical health problems at the same time. Moreover, it appeared that physical and mental health problems often occur together. For this reason, this study compares the association of on the one hand two physical health problems with work ability and on the other hand of one physical health problem and one or more mental health problems with work ability (*definition iii*).

Next, the interaction between physical and mental health problems is complex and has led to conflicting results in research on disability outcomes. Several studies have found a reinforcing interaction effect [24, 25]. This means that the effect of physical and mental health problems at the same time is greater than the sum of their separate effects. However, the opposite was also shown in another study where an attenuating interaction effect was found on role disability [26], meaning that the effect of their combination was smaller than the sum of their parts. These divergent findings could be explained by the fact that the researchers used either a linear regression with an additive scale or a logistic regression with a multiplicative scale to assess interaction effects [27, 28]. The use of a logistic regression is more likely to result in no interaction effect or a negative interaction effect as compared to the use of linear regression. An additive model is recommended for research into the combination of physical and mental health problems, as is applied in the current study [27]. It is likely that the results for disability are similar for work ability, as these concepts are closely related [29]. In this study the interaction-effect between mental and physical health problems on work ability will be researched (*definition iv*). In short, the first aim of this study is to research the association between the four operationalizations of multi-morbidity and work ability.

The effects of multi-morbidity on work ability may be countered by applying appropriate coping styles. By applying coping styles, attempts are made to face stressful situations, e.g., being confronted with physical and/or mental health problem(s) [30]. A distinction can be made between active, avoidant, and social support seeking coping styles. Through active coping, the stressful situation is addressed, whereas with avoidant coping contact with the stressful situation is avoided. In support seeking coping, help of significant others is sought [31]. Usually individuals apply the same coping style during different situations and stages of life [32], but it is possible to change coping styles [33, 34].

Research shows that active coping is related to better well-being and health, while avoidant coping is related to mental health problems and more physical symptoms [11–14]. Furthermore, it appears that physical and mental health problems lead to differences in productivity depending on the applied coping styles [15]. Moreover, research by van de Vijfeijke et al. using the dataset also used in the present study showed that active coping is associated with higher work ability and avoidant coping is related to lower work ability. In the van de Vijfeijke et al. study the role of coping style in the association between mental health and work ability and physical health and work ability was assessed. Van de Vijfeijke et al. did not focus on multi-morbidity; the researchers did not examine the role of coping style when multiple health problems are present, nor when physical health problems as well as mental health problems are present at the same time [35].

Limited research has been done on the associations between multi-morbidity, coping and work ability, though it has been recommended to not study coping styles of employees separately, but instead in combination with health problems and associated consequences [36]. Research about the role of coping styles in the relation between multi-morbidity and work ability of employees is relevant, because it may provide insights in the reason why ageing individuals are able to keep working, despite having multiple health problems. Therefore, it may provide opportunities for work place interventions. The first aim of the current study was thus to research the relationship between multi-morbidity and work ability (aim 1). To determine whether the effects of multiple health problems on work ability are mediated by coping styles, the second aim of this study is to explore whether health problems are related to coping styles and whether coping styles are in turn related to work ability (aim 2).

## Methods

In the current study data from the Study on Transitions in Employment, Ability and Motivation (STREAM) was used. STREAM is a longitudinal cohort study with three years of follow up (2010–2013). In STREAM, Dutch respondents aged 45–64 at baseline completed an annual online questionnaire on various topics such as health, employment status, and work ability. The current 1-year longitudinal study used the two most recent waves available, i.e. 2012 (wave 3) and 2013 (wave 4). In the first 2010 measurement, 15,118 individuals participated in the STREAM questionnaire, of which 10,363 participated in 2013. Since this study focuses on work ability, non-employed participants were excluded (remaining n = 7204). Further, participants with missing information on relevant concepts were excluded, resulting in a sample of 7175 participants.The Free University of Amsterdam Medical Ethics Committee declared that the Medical Research Involving Human Subjects Act does not apply to the STREAM and raised no objections to the execution of this research. In the information that accompanied the online questionnaire it was emphasized that privacy was guaranteed, that all answers were anonymous and would be treated confidentially and that all data were stored on secured computer systems.

### Measures

#### Work Ability

Work ability was assessed in both waves using the question: “If you would rate your work ability in the best time of your life at 10 points, at how many points would you rate your work ability at this moment?”. In this first item of the Work Ability Index (WAI) respondents assess their current work ability compared to the best time of their life using a scale from zero to ten, wherein zero represents very poor and ten represents lifetime best. The first item of the WAI is closely related to the overall WAI [37, 38]. Work ability at wave 1 and wave 2 were correlated (Pearson r = 0.74, p < 0).

#### Multi-morbidity

The occurrence of health problems at both waves was assessed with the question: “Do you currently have one or more of the following chronic diseases, disorders or handicaps?” [39]. Thirteen answer options were presented. Subsequently, two categories of health problems, i.e., physical and mental, were created. The category physical health problems was created by aggregating the answer options musculoskeletal disorders, severe headache or migraines, severe skin diseases, hearing problems, vision problems, epilepsy, circulatory, respiratory, and digestive health problems. The other category, mental health problems, includes the answer option “psychological health problems”. Throughout the article we refer to ‘one or more mental health problem(s)’ because this single answer category does not make it possible to distinguish persons with one mental health problem from those with multiple mental health problems.Four different operationalizations of mental and physical (multi-)morbidity were used in this study. The reference category for each operationalization was ‘no health problems’. First, the number of health problems was defined as *definition (i) S*econd, *only* mental health problem(s) or *only* physical health problem(s) were defined in *definition (ii*) Third, two physical health problems or one physical and one or more mental health problem(s) were distinguished for *definition (iii*) Lastly, the effects of one physical health problem or one or more mental health problem(s) were defined in *definition iv*; in this definition it does not matter whether the other type of health problem was present (as compared to *definition ii* where exclusivity was the case). Using this last definition, an interaction-effect between mental and physical health problems was researched.

### Coping Style

Coping styles were measured using nine items derived from the Utrecht Coping List (UCL), assessing to which degree respondents use active, avoidant or social support seeking coping styles. Three items were used for each coping style (Cronbach’s alpha active 0.77, avoidant 0.73, support seeking 0.70). The nine items were measured using a four-point Likert scale (1 = rarely or never; 4 = very often). For every participant an average score for each coping style was calculated.

### Covariates

Gender, age, educational level, and work status were included in all analyses as covariates. Age was stratified into five-year age groups (i.e., 45–49, 50–54, 55–59, 60–64 in the first STREAM wave). Educational level was assessed using the question: “What is your highest achieved education?”, the answers were grouped into three levels i.e., low, middle and high. Lastly, self-reported work status was grouped into two levels, i.e., company-employed and self-employed.

### Statistical Analysis

Descriptive statistics were used to report on the characteristics of the sample. Multiple linear regression analyses were conducted to assess the influence of multi-morbidity on work ability. The four different operationalizations of multi-morbidity were used for these analyses. For each operationalization, the reference category was ‘no health problems’. First, the influence of the number of health problems (*definition i*) at baseline (wave 1; 2012) on work ability at follow-up (wave 2; 2013) was assessed. Second, the influence of *only* mental and *only* physical health problems (*definition ii*) on work ability was determined. Next, the effect of two physical health problems and one physical and one or more mental health problem(s) (*definition iii*) on work ability was determined. Lastly, the effects of one physical health problem or one or more mental health problem(s) was analyzed (*definition iv*). Using this last definition, an interaction effect between physical and mental health problem(s) on work ability was examined. In order to create a product-term of one physical and one or more mental health problem(s), centered variables for mental and physical health problems were created by subtracting their means. By using this centering methodology multicollinearity is reduced [40].

**Fig. 1:**
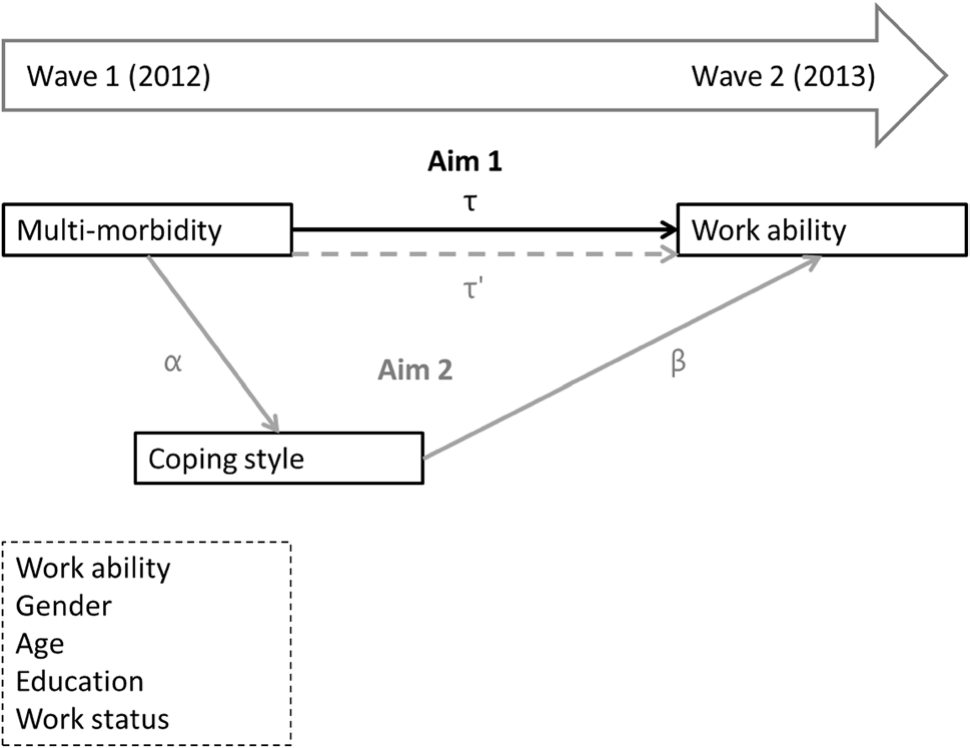
Schematic overview of research questions and analyses

To address the second aim and test whether coping styles mediate the influence of multi-morbidity on work ability, Sobel tests were performed separately for each of the three coping styles. These analyses focused on the operationalization of multi-morbidity defined as two physical health problems or one physical and one or more mental health problem(s) (*definition iii*). First, using multiple linear regressions, we determined the unstandardized regression coefficient and standard error for the association between multi-morbidity and the mediator, i.e., the three coping styles at baseline (Fig. 1; arrow α). Next, we determined the unstandardized regression coefficient and standard error for the association between the mediator at baseline (i.e., the three coping styles) and work ability at follow-up (Fig. 1; arrow β). Hereafter, we determined the extent of mediation using a Sobel test. To do so, we determined the percentage of the total effect (Fig. 1; arrow τ′) that was mediated by the coping styles using the formula [αβ/(αβ + τ′)]. In this formula τ′ is the coefficient for the association between the independent variable (i.e., multi-morbidity) and the outcome at follow up (i.e., work ability), adjusted for the mediator (i.e., coping style) [41]. Software by Preacher and Leonardelli [42] was used to perform this test.

All analyses were adjusted for work ability at baseline and the covariates. By adjusting for baseline work ability we took account of differences in work ability at baseline, which contributes to ruling out reversed causality [43]. For all analyses, unstandardized regression coefficients (B), their standard error (SE), and statistical significance (*p*) are reported. For all statistical analyses Statistical Package for the Social Science 23 (SPSS) was used.

## Results

### Sample Characteristics, Frequencies and Descriptives

Descriptive information about the sample can be found in Table 1. Most participants were between 55 and 59 years old. Less than half of the study population (43.3%) had no health problems, 31.4% had one health problem and 17.1% had two (*definition i*). More than half of the population had only physical health problems (53.3%), and only a small portion had only mental health problems (0.8%) (*definition ii*). 15.9% of the sample had two physical health problems, and 1.3% of the sample had one physical and one or more mental health problem(s) (*definition iii*). Only 3.4% of the sample had one or more mental health problem(s) (regardless of whether they also had a physical health problem), whereas 31.8% had one physical health problem (regardless of whether they also had a mental health problem) (*definition iv*). Within the group with one or more mental health problem(s) many persons also had a physical health problem (76.3%) (*definition iv*). On the other hand, only 4.7% of persons with a physical health problem also had a mental health problem (*definition iv*). At wave 1 (2012) the mean score of work ability was 7.93 and at follow-up (2013) this was 7.89.

**Table 1:** Characteristics of the study population at wave 1 (2013)

	n (%) Mean (SD)
Covariates	
Gender	
Males	4061 (56.6%)
Females	3114 (43.4%)
Age (2010)	
45–50	1956 (27.3%)
50–54	2094 (29.2%)
55–59	2172 (30.3%)
60–64	953 (13.3%)
Education	
Low	1820 (25.4%)
Medium	2779 (38.7%)
High	2576 (35.9%)
Work status	
Employee	6542 (91.2%)
Self-employed	633 (8.2%)
Work ability (range 0–10)	
(Wave 1, 2012)	7.93 (1.49)
(Wave 2, 2013)	7.89 (1.49)
Multi-morbidity	
Number of health problems *(definition i)*	
0 Health problems	3108 (43.3%)
1 Health problem	2250 (31.4%)
2 Health problems	1230 (17.1%)
3 Health problems	416 (5.8%)
4 Health problems	129 (1.8%)
5 Health problems	42 (0.6%)
Only physical or only mental *(definition ii)*	
Only physical health problem(s)	3822 (53.3%)
One or more mental health problem(s)	58 (0.8%)
Multiple physical and/or mental health problems *(definition iii)*	
Two physical health problems	1139 (15.9%)
One physical and one or more mental health problem(s)	91 (1.3%)
Mental and physical health problem(s) *(definition iv)*	
One or more mental health problem(s)^a^	245 (3.4%)
One physical health problem^a^ (at least)	2283 (31.8%)
Coping styles (range 1–4)	
Active	2.91 (0.55)
Avoidant	1.73 (0.49)
Social support seeking	2.18 (0.55)

% percentage of the whole sample; *SD* standard deviation; N = 7175

^a^Used in creating the interaction term

Employees lost to follow-up between wave 1 and wave 2 did not statistically significantly differ with regard to wave 1 multi-morbidity, work ability, or coping style as compared to employees that were not lost to follow-up (all *p* > 0.05). All covariates were related to at least one of the main concepts of the analyses and were therefore included in all analyses (results not shown).

### Multi-morbidity and Work Ability

Table 2 shows that a higher number of health problems was related to poorer work ability (model 1). When one health problem was used as the reference category, two health problems was related to a statistically significantly poorer work ability. When two health problems was used as reference, three health problems was related to a statistically significantly poorer work ability. However, having four health problems as compared to three was no longer related to a statistically significantly poorer work ability, nor was five compared to four.

**Table 2:** Association between multi-morbidity and work ability

	B	SE	Adj. R^2^
Model 1: *definition i*			20.30%
1 Health problem	− 0.18**	0.04	
2 Health problems	− 0.42**	0.05	
3 Health problems	− 0.62**	0.07	
4 Health problems	− 0.66**	0.12	
5 Or more health problems	− 0.63**	0.21	
Model 2: *definition ii*			19.40%
Only physical health problem(s)	− 0.24**	0.03	
Only mental health problem(s)	− 0.52**	0.19	
Model 3: *definition iii*			19.30%
2 Physical health problems	− 0.24**	0.04	
1 Physical and 1 or more mental health problem(s)	− 0.71**	0.14	
Model 4a: *definition iv*			18.60%
1 Physical health problem	− 0.17**	0.03	
1 Or more mental health problem(s)	− 0.68**	0.11	
Model 4b: *interaction effect*			18.50%
1 Physical health problem—centered	− 0.17**	0.03	
1 Or more mental health problem(s)—centered	− 0.65**	0.10	
*Interaction term*	− 0.11	0.21	

Reference category in all models is ‘no health problems’; dependent variable is work ability at wave 2; covariates included in each model: gender, age, educational level, work status, and work ability at wave 1

*B* unstandardized regression coefficient; *SE* standard error; *Adj. R*^*2*^ adjusted R^2^ (explained variance)

*p < 0.05; **p < 0.01; N = 7175

Furthermore, Table 2 shows that physical and mental multi-morbidity, operationalized in four different ways, was related to lower work ability at follow-up. Having mental health problem(s) was related to much poorer work ability than having physical health problem(s) (model 2 and model 4a). Furthermore, the effect of two physical health problems (B = − 0.24**) on work ability was much weaker than the effect of one physical and one or more mental health problem(s) (B = − 0.71**) (model 3). When having two physical health problems was used as reference category instead of the reference category of having no health problems, we also saw that one physical and one or more mental health problems was related to poorer work ability (B = − 0.56**; results not presented in Table 2).

**Fig. 2:**
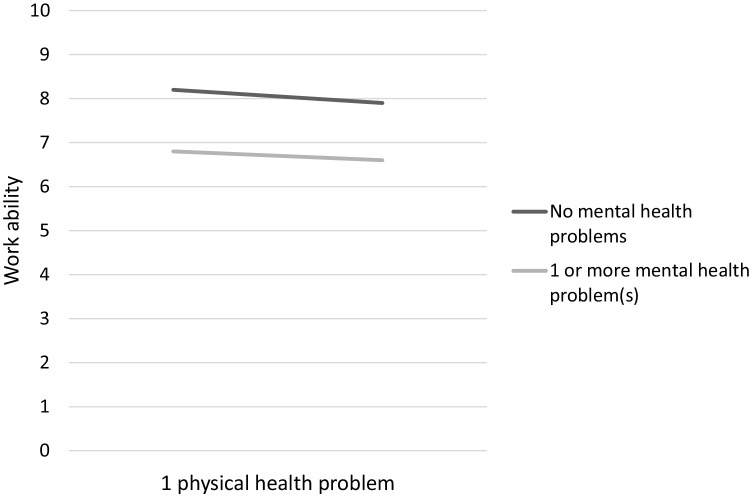
Figure of the interaction effect between one physical and mental health problem(s)

The interaction effect between one physical and a mental health problem was not significant (model 4b), which means that the negative effect on work ability of the combination of one physical and mental health problems was not significantly greater or smaller than the sum of their parts. Figure 2 in the Appendix shows two relatively parallel lines, which exemplifies this finding.

### The Role of Coping Style in the Multi-morbidity–Work Ability Association

Table 3 shows the mediating effect (αβ) of coping styles in the multi-morbidity–work ability association. For these analyses the third operationalization of multi-morbidity was used (*definition iii*), in which employees with two physical health problems, one physical and one or more mental health problem(s), and without health problems were compared. The mediation effect is the product of the coefficient for the association of the independent variable (i.e., multi-morbidity) and the mediator (α) (i.e., coping style) and the coefficient for the association of the mediator and the outcome variable (β) (i.e., work ability). Next, the direct effect (τ′) is shown, which is the coefficient for the independent variable and the outcome, adjusted for the mediator. Finally, the extent of mediation is presented, expressed as the percentage of mediation of the total effect [αβ/(αβ + τ′)].

**Table 3:** Mediation effects of coping style on the multi-morbidity–work ability association

Independent variable	Α	Β	τ′ (direct)	αβ (indirect)	αβ/(αβ + T′)
Dependent variable	Coping	WA	WA	Sobel-test	
Model 1: active coping *definition iii*					
2 Physical health problems	0.06 (0.02)**	–	− 0.25 (0.04)**	0.01**	− 0.04 (4%)
1 Physical and 1or more mental health problem(s)	− 0.19 (0.06)**	–	− 0.69 (0.14)**	–0.03**	0.04 (4%)
*Active coping*	–	0.14 (0.03)**	–	–	–
Model 2: avoidant coping *definition iii*					
2 Physical health problems	0.01 (0.02)	–	− 0.24 (0.04)**	− 0.002	0.001 (0.1%)
1 Physical and 1 or more mental health problem(s)	0.34 (0.05)**	–	− 0.67 (0.14)**	− 0.05**	0.07 (7%)
*Avoidant coping*	–	− 0.17 (0.03)**	–	–	–
Model 3: social support seeking *definition iii*					
2 Physical health problems	0.04 (0.02)*	–	− 0.24 (0.04)**	0.002	− 0.008 (0.8%)
1 Physical and 1 or more mental health problem(s)	− 0.08 (0.06)	–	− 0.71 (0.14)**	− 0.004	0.006 (0.6%)
*Social support seeking* coping	–	0.05 (0.03)	–	–	–

Reference category in all models is ‘no health problems’; covariates gender, age, educational level and work status and work ability at wave 1 included in all models

*α* unstandardized regression coefficient (with standard error in brackets) for the association between the independent variable and the active, avoidant and support seeking coping styles, *B* unstandardized regression coefficient (standard error) of the association between the active, avoidant and support seeking coping styles and work ability at wave 2, *τ′* the direct effect = unstandardized regression coefficient (standard error) t of the association between the independent variable and work ability at wave 2 adjusted for coping styles, *αβ* the indirect effect (A * B), *αβ/(αβ + τ′)* the extent of mediation, *WA* work ability

*p < 0.05; **p < 0.01; N = 7175

The first column (α) in Table 3 shows that employees with one physical and one or more mental health problem(s) less often used active coping (B = − 0.19**) and more often used avoidant coping (B = 0.34**) than employees without health problems. There was no significant difference for support seeking coping (B = − 0.08). Employees with two physical health problems did not differ from employees without health problems with regard to the use of avoidant coping, and slightly more often used active (B = 0.06**) and support seeking (B = 0.04*) coping.

The second column (β) in Table 3 shows that active coping had a positive association with work ability (B = 0.14**), avoidant coping had a negative association with work ability (B = − 0.17**) and support seeking coping did not have a significant association with work ability (B = 0.05).

The third column (τ′ direct) in Table 3 shows that employees with two physical health problems, when adjusting for active coping, had a poorer work ability than employees without health problems (B = − 0.25**). When comparing this direct, or non-mediated, association to the total effect (τ, not adjusted for coping style), as presented in Model 3 in Table 2 (B = − 0.24**), the direct effect was slightly stronger. This is subsequently reflected in the statistically significant Sobel test (fourth column—αβ) of 0.01**. Four percent of the negative total effect of physical health problems on work ability was indirectly suppressed via active coping (fifth column in Table 3).

The association between two physical health problems and work ability was unchanged when adjusting for avoidant and support seeking coping as compared to when not adjusting for these coping styles (i.e., B = − 0.24**). There was thus no mediation effect by these coping styles, as was also reflected in the non-significant Sobel test and small percentage of the total effect being mediated by these coping styles.

For one physical and one or more mental health problem(s) mediation was seen for both active and avoidant coping. For active coping, the direct, or non-mediated effect (τ′) of one physical and a mental health problem on work ability was weaker than the total effect (τ) (B = − 0.69 and − 0.71, respectively). The Sobel test showed a statistical significant mediation of − 0.03**; 4% of the total negative effect is indirectly increased via lowered active coping.

For the combination of one physical and one or more mental health problem(s) the direct, or non-mediated effect (τ′) on work ability when adjusting for avoidant coping was weaker than the total effect (τ) (B = − 0.67 and  − 0.71, respectively). The Sobel test showed a statistical mediation of − 0.05%, which means that seven percent of the total negative effect of physical and mental health problems on work ability was indirectly increased via avoidant coping. The seeking social support coping did not mediate the association between one physical and mental health problems and work ability.

## Discussion

This study shows that ageing employees with more health problems have poorer work ability, but that this negative effect stabilizes at three or more health problems. Furthermore, it appears that mental health problems have a stronger negative effect on work ability than physical health problems, both in combination with a physical health problem as well as when they are solely present. No significant interaction effect was found between one physical health problem and one or more mental health problem(s). The latter implies that the effect of the combination of one physical and one or more mental health problem(s) on work ability is not significantly greater or smaller than the sum of their parts.The negative association between multi-morbidity in terms of two physical health problems and work ability is partly reduced by the more active coping styles used in these ageing employees. Avoidant and support seeking coping styles do not play a significant role in the association between two physical health problems and work ability. The negative association between multi-morbidity in terms of physical and mental health problems and work ability can be partly explained by the use of less active coping as well as more avoidant coping. Support seeking coping does not have a role in the negative association between a physical and mental health problems and work ability.

The found negative association between the number of health problems and work ability supports our expectations and the literature. Recent research shows that a higher number of diseases is associated with poorer quality of life [44]. Quality of life is in turn strongly associated with work ability [45]. The detected stronger negative effect of mental health problems as compared to physical health problems with work ability is also consistent with expectations. Literature shows that mental health problems compared to physical health problems result in a greater loss of work ability, work productivity, work functioning [46] and work days [5].

No significant interaction effect was found between one physical and one or more mental health problem(s). Previous research has shown conflicting results about the interaction effects between mental and physical health problems. Both reinforcing and weakening interaction-effects have been found [24–26]. The lack of a significant interaction effect between mental and physical health problems in the current study could possibly be explained by interaction effects in different directions that occur at the same time. The possibility exists that interaction effects compensate for each other in the case that weakening as well as reinforcing interaction effects are concurrently present. The result is that no significant interaction effect can be noticed. The current study showed that individuals with two physical health problems used more active coping, which partly countered their lower work ability compared to healthy individuals. Furthermore, no significant mediation by avoidant coping in the physical multi-morbidity–work ability association was found in this study. On the other hand, individuals with both physical and mental health problems used less active coping, which partly explained their lower work ability compared to those without health problems. Ageing employees with both physical and mental health problems were more likely to use avoidant coping, which partly explained why their work ability was lowered. The negative association between one physical and one or more mental health problem(s) and work ability was partly mediated by avoidant coping.Active coping appeared to have a positive effect on work ability in this sample, which is in line with several other studies examining different work outcomes [12, 14, 15]. The negative effect of avoidant coping has also been shown in other studies [12, 14]. The lack of a mediating effect of this coping style on the association between two physical health problems and work ability could be due to the fact that individuals with two physical health problems did not use this avoidant coping style more often than healthy individuals; statistically significant relations between the independent, mediator, and dependent variable with one-another are a prerequisite to finding mediation effects. This study also showed that support seeking coping does not mediate the association between multi-morbidity and work ability. These findings are consistent with a similar study concerning health problems, coping styles and work ability done with the same dataset as used in the current study [35]. A possible explanation for the lack of effect of social support seeking coping on work outcomes is given by Van Rhenen et al. [14]. These authors argue that social support seeking coping could reduce sickness absence, but that social support on the contrary could advocate absence-related performance and encourage a person to stay at home when dealing with health problems. Work outcomes thus may be influenced in two contrary ways, which could cancel out any effects.The main strengths of this study include its large and diverse sample (in terms of professional background and health problems) and longitudinal character. However, some potential limitations should be mentioned. First of all, it is unclear how many health problems an individual has within the category of mental health problems, e.g., persons with one or with two or more mental health problems are grouped together. Further, we focused on health problems as measured at wave 1 and did not explore whether the health problems were present at follow-up. These two aforementioned limitations could have caused heterogeneity in our analysis. Furthermore, the severity of the health problem was not included in the analyses. Previous research has shown that correcting for severity of the disease results in a stronger negative association between multi-morbidity and quality of life, which is closely related to work ability [45, 47]. Furthermore, we only made a distinction between physical and mental health problems, but we did not distinguish the specific type of health problem within these categories. In addition, only 3.4% of the sample indicated that they suffer from a mental health problem. This prevalence is lower than shown in another Dutch study [48]. This is potentially due to the so-called healthy worker effect, whereby ageing workers may drop out of the workforce due to health problems, which in turn may result in healthier remaining workers [49]. Next, multi-morbidity is measured in multiple ways in the literature and there is a lack of consensus on the best measure. However, research shows that different measures of multi-morbidity lead to divergent health related outcomes [50]. Furthermore, we adjusted for baseline work ability to partially rule out reversed causality. But to do this wholly, the reverse effects should be examined. Research shows that it is possible that coping styles predict work ability, which could in turn be mediated by health problems [51]. However, there is also evidence that mental and physical health problems are associated with work ability, which in turn is mediated by coping styles. Research about the reverse effects of the variables would provide more clarity about their predicting or mediating effects. Lastly, in future research the association between coping styles and work-related outcomes for non-working individuals should be explored to obtain more clarity about the role of coping resources for unemployed people.

In conclusion, this study provides novel insights into the associations between multi-morbidity, coping styles and work ability. Our findings confirm earlier research that mental health problems relate to a greater loss of work ability than physical health problems, both in combination with physical health problems as well as when they occur separately. Generally, employees with combined physical and mental health problems more often use unfavorable coping styles, which are in turn harmful for work ability. To prevent a loss in work ability and ultimately loss of employment, promoting favorable coping styles amongst employees with health problems should be seen as a key focus of work place health interventions.
